# The restless retina in aggressive posterior retinopathy of prematurity: prevention is better than cure

**Published:** 2018

**Authors:** Parijat Chandra, Ruchir Tewari, Shreyans Jain

**Affiliations:** Additional Professor of Ophthalmology: Dr Rajendra Prasad Centre for Ophthalmic Sciences, All India Institute of Medical Sciences, New Delhi, India.; Senior Resident (Ophthalmology): Dr Rajendra Prasad Centre for Ophthalmic Sciences, All India Institute of Medical Sciences, New Delhi, India.; Senior Resident (Ophthalmology): Dr Rajendra Prasad Centre for Ophthalmic Sciences, All India Institute of Medical Sciences, New Delhi, India.

**Figure:**
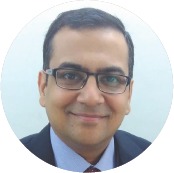
Parijat Chandra

**Figure:**
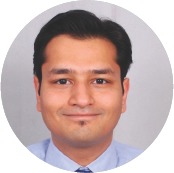
Ruchir Tewari

**Figure:**
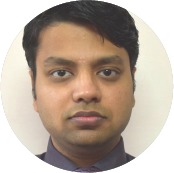
Shreyans Jain

**Aggressive Posterior Retinopathy of Prematurity (APROP) is a severe variant of ROP that usually affects the smallest and sickest babies. Following the best neonatal care practices is a good way to prevent it.**

**Figure 1:**
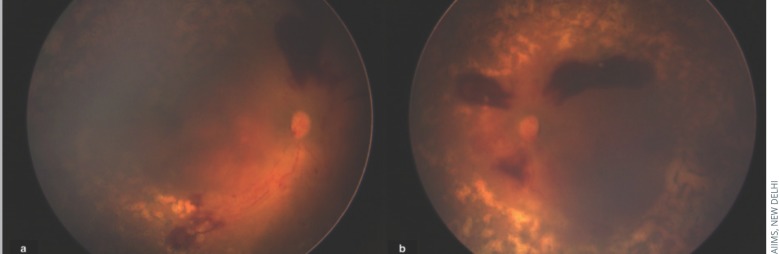
Fundus images of the right (a) and left (b) eyes showing very small zone 1 ROP with poor vascularised macula, plus disease, sub-hyaloid hemorrhages and peripheral laser spots.

A 34 weeks-old male baby with history of bilateral laser photocoagulation for zone 1 aggressive posterior retinopathy of prematurity (APROP) and disease progression was treated with bilateral intravitreal injection of half adult dose Bevacizumab (0.625mg/ 0.025ml). After an initial favorable response, the disease recurred and gradually progressed to stage 4A ROP with tractional retinal detachment (TRD) in both eyes necessitating surgical intervention. This case study highlights the relentless course of severe APROP, a disease which can be effectively prevented by good NICU practices.

## Introduction

Aggressive Posterior Retinopathy of Prematurity (APROP) is a severe variant of ROP that usually affects the smallest and sickest babies. It manifests as a relentless progressive retinopathy that occurs due to arrested vascularisation limited to very small areas of the retina (zone 1 or zone 2 posterior). This often leads to progressive retinal detachment despite treatment. APROP usually occurs due to poor neonatal care practices, notably exposure to unregulated oxygen for prolonged periods. Laser photocoagulation of the avascular retina has been the mainstay for ROP treatment, but recently anti-VEGF drugs have emerged as an alternative treatment in APROP, despite the safety concerns.

This case highlights the challenges associated with APROP, a disease which is difficult to control, and requires a multipronged treatment approach over a prolonged follow up period. The report stresses the need for better neonatal care which can prevent development of APROP and avoid the challenges for its treatment.

## Case Report

A male baby was born at 29 weeks gestational age with birth weight of 1100g. The baby's twin did not survive. The baby suffered from neonatal sepsis and apnea that required a stay at neonatal intensive care unit (NICU) for 33 days. Supplemental oxygen was given to the baby for a duration of 20 days including three days of continuous positive pressure ventilation in the NICU. Blood transfusions were performed twice. As per records, timely screening was performed at 32-week post menstrual age (PMA). The ophthalmologist noted a poorly dilating pupil, a small zone of vascularisation and presence of dilated and tortuous vessels at the posterior pole suggestive of zone 1 APROP. Uneventful laser photocoagulation of anterior avascular retina was performed using 532nm green laser, but the disease worsened and the child was referred to us for management.

**Figure 2:**
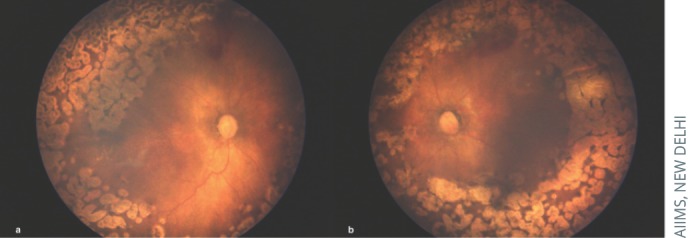
At PMA 41 weeks, good disease regression is observed in both eyes (a, b).

At 34 weeks PMA, the pupils were still poorly dilating. Fundus examination revealed a very small zone 1 APROP, with media haze and markedly dilated and tortuous vessels indicating severe plus disease with patches of sub-hyaloid bleed in both eyes (Figure 1). Intravitreal Bevacizumab (0.625mg/0.025ml – half adult dose) injections were given in both eyes under aseptic conditions in the same sitting in the operation theater pupillary dilation, improved media clarity, significant reduction in plus disease and partial resolution of the sub-hyaloid bleed. 7 weeks after injection (PMA 41 weeks), good disease regression was observed in both eyes (Figure 2).

**Figure 3:**
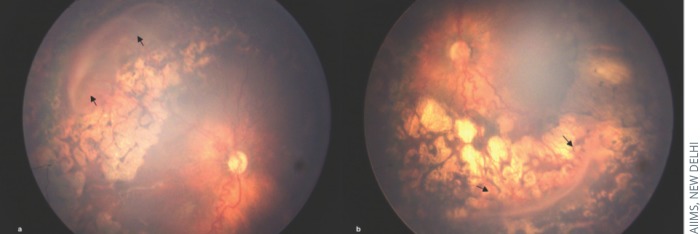
At 50 wks PMA, developing recurrence of plus disease with progressive FVP and TRD (black arrows) in the laser treated regions in both eyes (a, b).

**Figure 4:**
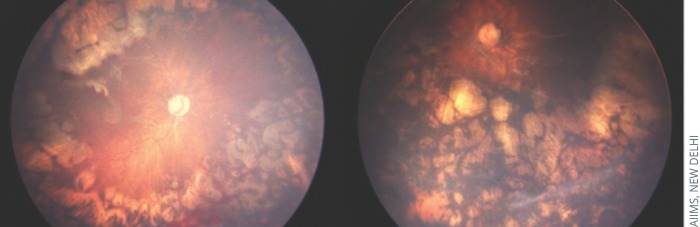
One month after LSV in both eyes, resolution of plus disease and regression of FVP is noted in both eyes (a, b).

However, at 48–50 weeks PMA, both eyes started developing dilation and tortuosity of vessels (plus disease) with progressive fibrovascular proliferation (FVP) and tractional retinal detachment (TRD) suggestive of stage 4A ROP (Figure 3). At 54 weeks PMA, bilateral 25G suture-less lens sparing vitrectomy (LSV) was performed. One month later, both eyes showed near complete disease regression (Figure 4). At the last follow up, the retina was attached with no evidence of retinal traction. The parents were counselled regarding a need for close and long term follow up for retinal status and detection of refractive errors.

## Discussion

This case study demonstrates the aggressive disease in APROP, a severe ROP variant which required treatment with laser, anti-VEGF drugs and surgery in both eyes, a prolonged follow up with active treatment over a period of months in a sick preterm baby.

### Emerging role of Anti-VEGF drugs in APROP

Laser treatment of avascular retina has been the gold standard for ROP treatment.^1^ However, laser treatment scars the avascular retina with limited prospects of further retinal growth. In small zone 1 ROP/APROP, this can lead to constriction of visual fields and high incidence of severe myopia. Anti-VEGF drugs like Bevacizumab/Ranibizumab are now emerging as a good alternative treatment in zone 1 ROP. The BEAT ROP^2^ study showed significantly better outcomes of Bevacizumab in cases of zone 1 ROP compared with laser, with decreased chances of recurrences and progression of normal retinal vascularisation, leading to future prospects of better visual fields.

Anti VEGF pharmacotherapy also has the added benefit of lesser incidence of high myopia,^3^ but is often complicated by delayed recurrences requiring much longer follow up, and laser treatment may be needed. The ideal dose for preterm babies, choice of Anti-VEGF drug, the role of multiple injections, and long-term safety (due to systemic absorption and VEGF suppression) still remains unclear due to lack of relevant data.^4^

Anti-VEGF agents are especially useful in cases with severe plus disease with rigid non-dilating pupils, where laser treatment is not possible. It leads to rapid pupillary dilation in a few days, that allows laser treatment to be completed. Currently, many use them as adjunct before or after laser treatment as well.

### Multimodal treatment for APROP

The diagnosis of zone 1 APROP needs a high index of suspicion because of a featureless vascular-avascular junction and avascular loops, which confuse the observer. Indeed, prompt aggressive treatment is warranted as the disease can progress rapidly. Multiple treatments are needed with laser or Anti-VEGF drugs, or a combination of both, but the outcome is unpredictable. Our case had delayed recurrence despite combined treatment, and quickly progressed to retinal detachment needing surgical treatment. However, timely surgical management in developing countries is still difficult due to lack of advanced vitreoretinal surgical setups; experienced paediatric retinal surgeons and; trained anaesthesia teams willing to provide general anaesthesia to such small babies. Such services are scarce and available in very few apex eye care facilities.

### ROP Prevention is better than cure

It is well known that implementation of best neonatal practices and simple measures like strict regulation of oxygen delivery can prevent development of severe ROP.^5^,^6^ In fact, absence of zone 1 APROP is considered an important marker of quality neonatal care. But poor NICU care across the country is leading to severe ROP developing in even larger babies.^7^ This has led to repeated changes in screening guidelines to include these bigger babies as well. Lack of proper screening and treatment facilities, adds to increase in the number of cases with ROP blindness. Thus, it is recommended to follow the best neonatal care practices to prevent ROP and especially APROP.
